# Identification of Differentially Expressed miRNAs and mRNAs in Vestibular Schwannoma by Integrated Analysis

**DOI:** 10.1155/2019/7267816

**Published:** 2019-06-11

**Authors:** Yanhua Lei, Ping Guo, Xiuguo Li, Yuanyuan Zhang, Ting Du

**Affiliations:** Department of Otolaryngology, Jining No. 1 People's Hospital, China

## Abstract

**Background:**

Vestibular schwannoma (VS) is benign, slow-growing brain tumor that negatively impacts patient quality of life, which may cause even death. This study aimed to explore key genes and microRNAs (miRNAs) associated with VS.

**Methods:**

The mRNA and miRNA expression profiles of VS downloaded from Gene Expression Omnibus (GEO) database were included in this study to perform an integrated analysis. The differentially expressed mRNAs (DEmRNAs) and miRNAs (DEmiRNAs) were identified. Then, functional annotation and protein-protein interaction networks (PPI) of DEmRNAs were constructed. DEmiRNA-target DEmRNAs analysis and functional annotation of DEmiRNA-target DEmRNAs were performed.

**Results:**

A total of 2627 DEmRNAs (1194 upregulated and 1433 downregulated DEmRNAs) and 21 DEmiRNAs (12 upregulated and 9 downregulated DEmiRNAs) were identified. ISG15, TLE1, and XPC were three hub proteins of VS-specific PPI network. A total of 2970 DEmiRNAs-DEmRNAs pairs were obtained. Among which, hsa-miR-181a-5p, hsa-miR-106-5p, and hsa-miR-34a-5p were the top three DEmiRNAs that covered most DEmRNAs. The functional annotation of DEmiRNA-target DEmRNAs revealed that the DEmiRNA-target DEmRNAs were significantly enriched in cGMP-PKG signaling pathway, adrenergic signaling in cardiomyocytes, and pathways in cancer.

**Conclusion:**

The results of this present study may provide a comprehensive understanding for the roles of DEmRNAs and DEmiRNAs in the pathogenesis of VS and developing potential biomarkers of VS.

## 1. Introduction

Vestibular schwannoma (VS), commonly termed acoustic neuromas, arise from the vestibular branch of the eighth cranial nerve and is benign, slow-growing brain tumors that negatively impact patients' quality of life, which may cause hearing loss, tinnitus, facial palsy, and when large enough, brain stem compression, and even death [1, 2]. VS may appear unilaterally but may also appear bilaterally when associated with neurofibromatosis type 2 syndrome (NF2) [2]. To date, the identification of the NF2 gene is the most important finding to our understanding of VS biology [3]. Current treatment modalities of VS are various, including observation, also known as wait-and-scan or watchful waiting, radiation therapy (RT) and microsurgical resection (MS), based on assorted factors, such as size at diagnosis, significant tumor growth on serial imaging, or patient symptoms [4].

Recent efforts to define the associated genes and molecular pathways involved in tumorigenesis and expansion have been met with some success. Welling et al. identified a number of deregulated genes in tumor tissue by using cDNA microarray analysis of tissue samples from 1 vestibular nerve versus 3 cystic sporadic, 3 solid sporadic, and 1 NF2-associated vestibular schwannoma [5]. Cayé-Thomasen et al. examined the gene expression in tissue samples from 3 human vestibular nerves versus 16 solid, sporadic vestibular schwannomas using a microarray chip and identified more than 20,000 genes [6]. Aarhus et al. studied 25 VSs and 3 tibial nerves (controls) with the ABI 1700 microarray platform and obtained 1650 differentially expressed genes [7]. However, the molecular events involved in the development of this condition are not well understood. There is an urgent need to investigate new therapeutic targets for VS and develop novel treatment options.

MicroRNA (miRNA) is a new class of noncoding RNA molecules, which is short (~21-23 nucleotides long), single-stranded RNA molecules and later shown to be a key part of posttranscriptional regulatory mechanisms of gene expression in diverse organisms [8, 9]. MiRNAs broadly participates in the regulation of protein translation and mRNA stability and are believed to play pivotal roles in a wide array of biological processes, including tumor development [10–12]. As alterations in miRNA expression levels have been linked with a variety of disease processes, focus on aberrant expression in pathological states has increased [13].

This present study performed an integrated analysis of miRNAs and mRNAs expression profiles of VS downloaded from Gene Expression Omnibus (GEO) database. The differentially expressed mRNAs (DEmRNAs) and miRNAs (DEmiRNAs) were identified. In addition, protein-protein interaction (PPI) network of DEmRNAs was conducted. DEmiRNA-target DEmRNAs analysis and functional annotation of DEmiRNA-target DEmRNAs were performed. In doing so, we hope this study could represent a new avenue for understanding the pathogenesis and developing potential biomarkers of VS.

## 2. Materials and Methods

### 2.1. Microarray Expression Profiling in GEO

The mRNA and miRNA expression profiles of patients with VS were downloaded from GEO database (http://www.ncbi.nlm.nih.gov/geo). The search strategy in the GEO datasets was as follows: (1) selected datasets should be mRNA/miRNA transcriptome data of the whole genome; (2) these data were derived from tumor tissues and adjacent nontumor tissues of patients with VS; (3) datasets were standardized or raw datasets. Three datasets of mRNA expression profiles, including GSE108524, GSE56597, and GSE39645, and two datasets of miRNA expression profiles, including GSE43571 and GSE24390, were included in this study (Tables 1 and 2).

### 2.2. Identification of DEmRNAs and DEmiRNAs in Patients with VS Compared with Normal Controls

MetaMA, an R package, is used to combine data from multiple microarray datasets, and we obtained the individual P-values. The Benjamini & Hochberg method was used to obtain multiple comparison correction false discovery rate (FDR). DEmRNAs and DEmiRNAs in VS compared to normal controls were obtained with FDR < 0.05 and FDR < 0.01, respectively. Hierarchical clustering analysis of DEmRNAs was conducted by using R package “pheatmap”.

### 2.3. Functional Annotation of DEmRNAs between Patients with VS and Normal Controls

Functional annotation, including Gene Ontology (GO) function and Kyoto Encyclopedia of Genes and Genomes (KEGG) pathway enrichment analyses of the DEmRNAs between patients with VS and normal controls, were performed by CPDB (http://cpdb.molgen.mpg.de/CPDB). FDR < 0.01 was set as the cut-off for significance.

### 2.4. PPI Network Construction

Top 50 up- and downregulated DEmRNAs were scanned with the Biological General Repository for Interaction Datasets (BioGrid, http://www.uniprot.org/database/DB-0184). In order to further explore the biological functions of the DEmRNAs, PPI network was then constructed by using Cytoscape software (version 3.6.1, http://www.cytoscape.org).

### 2.5. DEmiRNA-Target DEmRNAs Analysis

As miRNAs tend to decrease the expression of their target mRNAs, target genes from DEmRNAs that expressed inversely with that of miRNA were selected to subject to further investigation [14]. Firstly, the putative targeted DEmRNAs of DEmiRNAs were predicted by six bioinformatic algorithms (RNA22, miRanda, miRDB, miRWalk, PICTAR2, and Targetscan). Then, the confirmed targeted DEmRNAs of DEmiRNAs were obtained from miRWalk. Thirdly, the confirmed DEmiRNA-DEmRNA pairs were derived from miRWalk and the DEmiRNA-DEmRNA pairs recorded by ≥ 4 algorithms. Based on the obtained DEmiRNA-DEmRNA pairs, DEmiRNA-DEmRNA interaction networks between VS and normal controls were constructed by using Cytoscape software (http://www.cytoscape.org/).

### 2.6. Functional Annotation of DEmiRNA Targets

To further research the biological function of the target DEmRNAs of DEmiRNAs, GO and KEGG pathway analysis were performed by using CPDB (http://cpdb.molgen.mpg.de/CPDB). FDR < 0.01 was set as the cut-off for significance.

## 3. Results

### 3.1. DEmRNAs and DEmiRNAs between Patients with VS and Normal Controls

**Figure 1:**
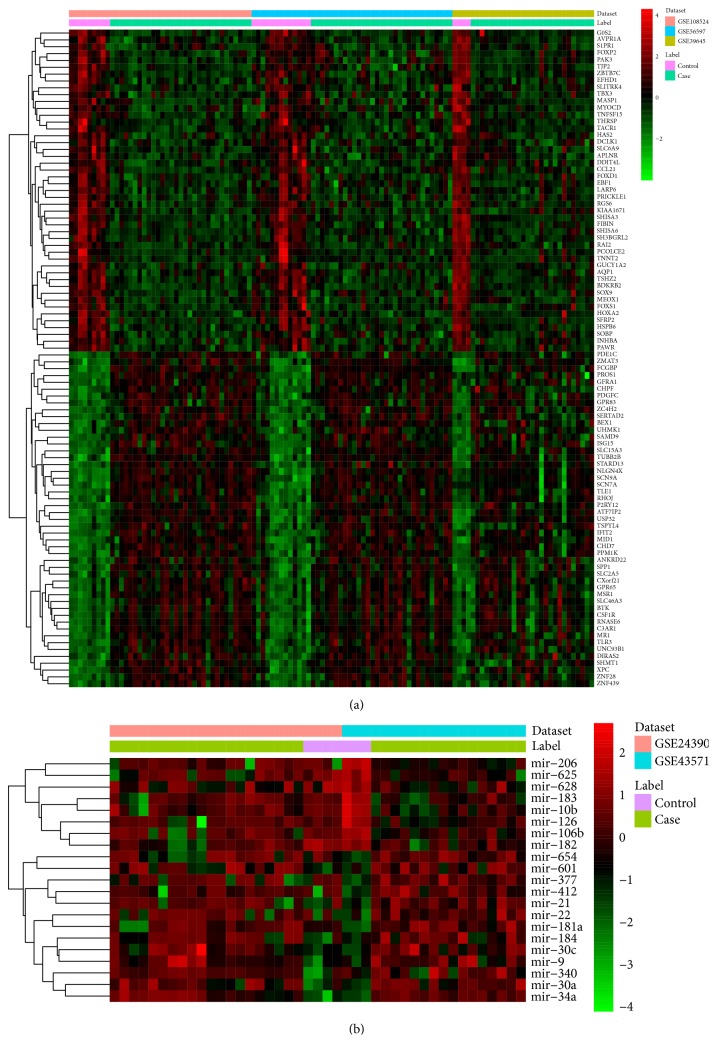
The heatmap of top 100 up- and downregulated DEmRNAs and DEmiRNAs between VS and normal controls. Row and column represented DEmRNAs/DEmiRNAs and tissue samples, respectively. The color scale represented the expression levels.

Compared to normal controls, a total of 2627 DEmRNAs (1194 upregulated and 1433 downregulated DEmRNAs) and 21 DEmiRNAs (12 upregulated and 9 downregulated DEmiRNAs) were identified. The top 10 up- and downregulated DEmRNAs and all DEmiRNAs between patients with VS and normal controls were displayed in Tables 3 and 4, respectively. Hierarchical clustering analysis of top 100 up- and downregulated DEmRNAs and DEmiRNAs was displayed in Figure 1.

### 3.2. Functional Annotation of DEmRNAs between Patients with VS and Normal Controls

**Figure 2:**
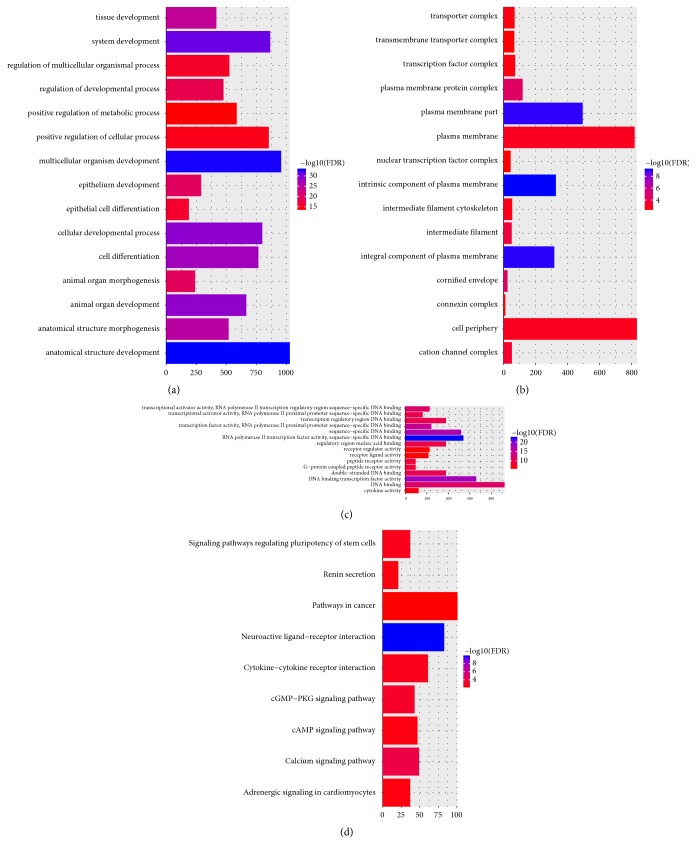
Significantly enriched GO terms and KEGG pathways of DEmRNAs between patient with VS and normal controls. (a). BP, biological process; (b). CC, cellular component; (c). MF, molecular function; (d) KEGG pathways. The x-axis shows counts of DEmRNAs enriched in GO terms or KEGG pathways and the y-axis shows GO terms or KEGG pathways. The color scale represented -lg FDR.

Anatomical structure development (FDR = 1.30E-33), multicellular organism development (FDR = 1.67E-33), intrinsic component of plasma membrane (FDR = 8.06E-10) and RNA polymerase II transcription factor activity, and sequence-specific DNA binding (FDR = 3.72E-23) were significantly enriched GO terms in VS (Figures 2(a)–2(c)). Neuroactive ligand-receptor interaction (FDR = 1.49E-10), calcium signaling pathway (FDR = 0.000137054), and cGMP-PKG signaling pathway (FDR = 0.000853812) were significantly enriched KEGG pathways in VS (Figure 2(d)).

### 3.3. PPI Network Construction

**Figure 3:**
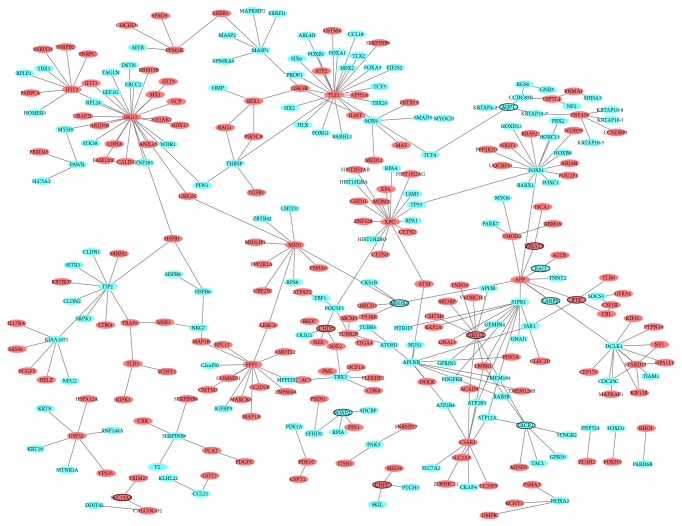
VS-specific PPI network. The red and green ellipses represented proteins encoded by up- and downregulated DEmRNAs between VS and normal controls. Ellipses with black border were DEmRNAs derived from top 10 up- and downregulated DEmRNAs between VS and normal controls.

The VS-specific PPI network was consisted of 308 nodes and 310 edges. ISG15 (degree = 26), TLE1 (degree = 24), and XPC (degree = 16) were three hub proteins of VS-specific PPI network (Figure 3).

### 3.4. DEmiRNA-Target Interactions

**Figure 4:**
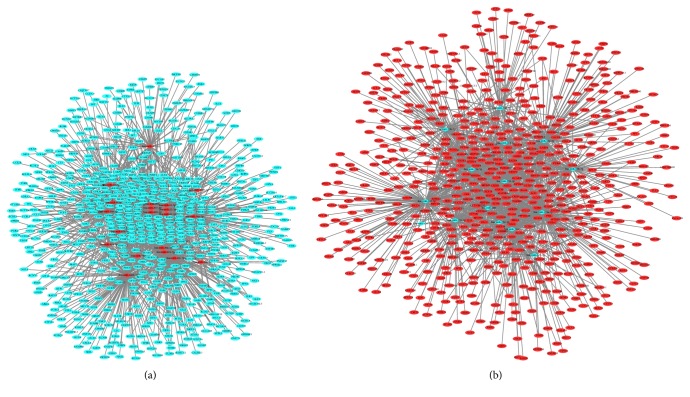
DEmiRNAs-DEmRNAs interaction network. (a) Interaction network between upregulated DEmiRNAs and downregulated DEmRNAs. (b) Interaction network between downregulated DEmiRNAs and upregulated DEmRNAs. The rhombic nodes and elliptical nodes indicate DEmiRNAs and DEmRNAs, respectively. Red and green color represent upregulation and downregulation, respectively.

A total of 2970 DEmiRNAs-DEmRNAs pairs, including 2570 DEmiRNAs-DEmRNAs pairs which were predicted by ≥ 4 algorithms and 568 validated DEmiRNAs-DEmRNAs pairs derived from the miRWalk, were obtained (Figure 4). Among which, hsa-miR-181a-5p (degree = 186), hsa-miR-106-5p (degree = 175), and hsa-miR-34a-5p (degree = 161) were the top three DEmiRNAs that covered most DEmRNAs.

### 3.5. Functional Annotation of DEmiRNA Targets

**Figure 5:**
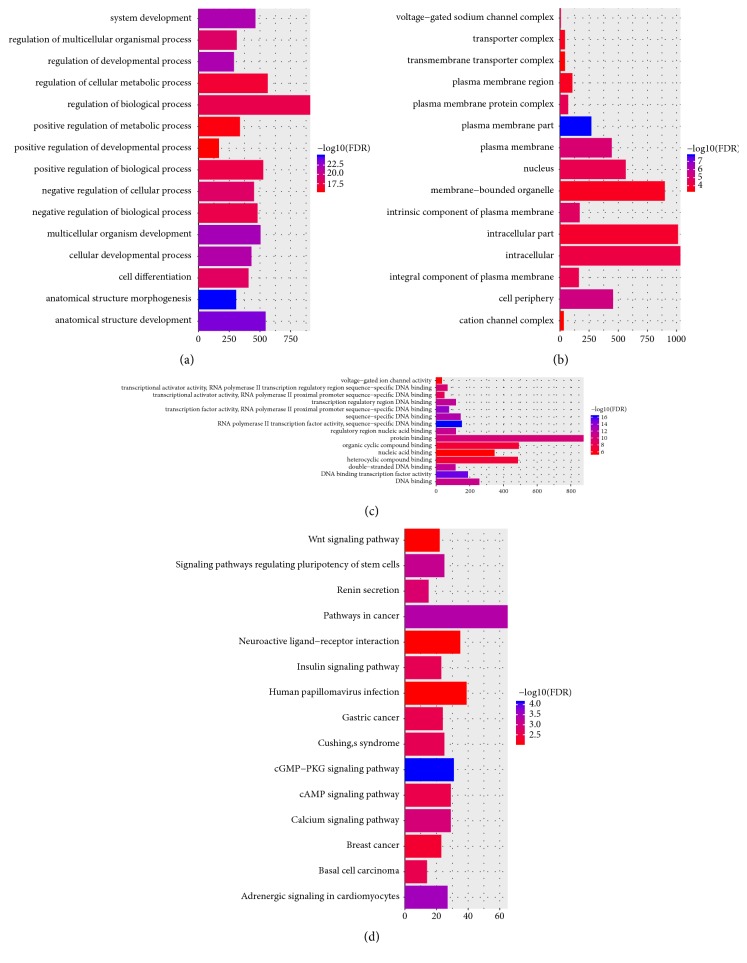
Significantly enriched GO terms and KEGG pathways of DEmiRNA-target DEmRNAs. (a). BP, biological process; (b). CC, cellular component; (c). MF, molecular function; (d) KEGG pathways. The x-axis shows counts of DEmRNAs enriched in GO terms or KEGG pathways and the y-axis shows GO terms or KEGG pathways. The color scale represented -lg FDR.

Based on GO enrichment analysis, anatomical structure development (FDR = 1.09E-25), anatomical structure development (FDR = 3.24E-24), plasma membrane part (FDR = 1.78E-08), and RNA polymerase II transcription factor activity, sequence-specific DNA binding (FDR = 5.25E-17) were significantly enriched GO terms in VS (Figures 5(a)–5(c)). According to the KEGG pathway enrichment analysis, the DEmiRNA-target DEmRNAs were significantly enriched in cGMP-PKG signaling pathway (FDR = 7.54E-05), adrenergic signaling in cardiomyocytes (FDR = 0.00029), and pathways in cancer (FDR = 0.000433) (Figure 5(d)).

## 4. Discussion

VS is a benign tumor originating from the nerve sheath of one of the vestibular nerves. It is the most common extra-axial tumor in the posterior fossa of adults, comprising over 80% of tumors in the cerebellopontine angle (CPA) [15]. In this study, we performed an integrated analysis based on the databases downloaded from GEO to obtain key mRNAs and miRNAs associated with VS. To the best of our knowledge, it is the first time to conduct an integrated analysis on miRNAs and mRNAs expression profiles of VS.

ISG15, a small molecular weight protein, whose expression is induced by interferon, was first identified as an ubiquitin-like modified protein and was named ubiquitin cross-reactive protein as its structure was similar to ubiquitin [16]. ISG15 has been postulated that it may directly or indirectly be involved in tumor development [17]. It was demonstrated that ISG15 was differentially expressed in different tumor cells and different cell lines from same histologic origin [18]. Zhang et al. reported that the expression of ISG15 mRNA and protein was significantly higher in tumors than in adjacent control tissues [19]. Reportedly, ISG15 and UBE2L6 were identified as negative regulators of autophagy in esophageal cancer cells [20]. In this present study, ISG15 was significantly upregulated and a hub gene in PPI network in VS. It is surmised that ISG15 may involve in VS.

TLE family member 1, transcriptional corepressor (TLE1), exhibits a well-characterized function in the regulation of nervous system development [21]. In particular, TLE1 exhibits antineurogenic activity in mammalian forebrain development [21]. TLE1 is involved in diverse signaling pathways and has important roles in neurogenesis, sex determination, and segmentation during development [22]. Previous studies suggest TLE1 could be used as a diagnostic marker and is a possible therapeutic target in various malignancies. Yao et al. reported that TLE1 was overexpressed in human lung tumors and may play an important role in promoting lung tumorigenesis and then speculated it may be a putative lung-specific oncogene [21]. Bakrin et al. suggested that TLE1 immunohistochemistry for synovial sarcoma can be very useful to distinguish synovial sarcoma from histological mimics [23]. Additionally, TLE1 deficiency resulted in enhanced tumor growth [24]. In this analysis, TLE1 was a hub gene in PPI network as well, which may imply the important role of TLE1 in VS.

Prickle1 is important for the nervous system development, and believed to be an integral part of the planar cell polarity (PCP) pathway [25]. Prickle1 has been shown to regulate neuron morphogenesis, including neuron migration and neurite growth in the mouse [25–27]. Yang et al. suggested that in mice, Prickle1 was part of a molecular mechanism that regulated facial branchiomotor neuron caudal migration and separated the facial branchiomotor neuron and the olivocochlear efferents [25]. Prickle1 was showed to be highly expressed in the spiral ganglion and involved in regulating distal and central outgrowth of spiral ganglion neuron neurites of the inner ear [28]. PRICKLE1 was detected to be one of top 10 downregulated DEmRNAs in VS. In view of this, we speculated PRICKLE1 may implicate in the pathogenesis of VS by participating in neuron morphogenesis.

Galanin is a multifunctional neuropeptide initially identified from the porcine intestine [29]. In mammals, galanin is widely distributed in the central nervous system and peripheral tissues, where it is involved in the modulation of hormone and neurotransmitter release, cognitive functions, and neuronal development [30]. The diverse physiological effects of galanin are mediated by at least three galanin receptor subtypes, including GalR1, GalR2, and GalR3 [30].

Misawa et al. indicated that GALR1/2 methylation status may serve as an important site-specific biomarker for prediction of clinical outcome in patients with head and neck squamous cell carcinoma [31]. In addition, according to the KEGG pathway enrichment analysis, GALR1 was enriched in neuroactive ligand-receptor interaction. These findings may show that GALR1 is associated with VS.

Based on the obtained DEmiRNAs-DEmRNAs pairs, they revealed that both PRICKLE1 and GALR1 are targets of hsa-miR-30c-5p and hsa-miR-30a-5p, which imply the key role of hsa-miR-30 in VS. Accordingly, we proposed a hypothesis that hsa-miR-30 may involve in VS by regulating PRICKLE1 and GALR1.

In conclusion, abundant DEmiRNAs and DEmRNAs between VS and normal controls were identified which may make a contribution for developing new diagnostic and therapeutic strategies for VS and emphasized the importance of several mRNAs and miRNAs which may implicate in VS. These findings may provide new insight into understanding the mechanism of VS. The exact function of these mRNAs and miRNAs in VS need to be determined with further research.

## Figures and Tables

**Table 1:** List of mRNA study samples from GEO.

GEO accession	Author	Platform	Samples (N:P)	Year
GSE108524	Zhao Fu	GPL17586 [HTA-2_0] Affymetrix Human Transcriptome Array 2.0 [transcript (gene) version]	4 : 27	2018
GSE56597	Miguel Torres-Martin	GPL10739 [HuGene-1_0-st] Affymetrix Human Gene 1.0 ST Array [probe set (exon) version]	9 : 30	2014
GSE39645	Miguel Torres-Martin	GPL6244 [HuGene-1_0-st] Affymetrix Human Gene 1.0 ST Array [transcript (gene) version]	9 : 31	2013

**Table 2:** List of miRNA study samples from GEO.

GEO accession	Author	Platform	Samples (N:P)	Year
GSE43571	Miguel Torres-Martin	GPL8786 [miRNA-1] Affymetrix Multispecies miRNA-1 Array	3 : 16	2014
GSE24390	Okay Saydam	GPL7436 Wurdinger/Krichevsky miRNA array IV	4 : 20	2010

**Table 3:** Top 10 up- and downregulated DEmRNAs between patients with VS and normal controls.

Gene symbol	ID	FDR	Regulation
LARP6	55323	0	down
PRICKLE1	144165	0	down
RAI2	10742	0	down
G0S2	50486	0	down
AQP1	358	0	down
MEOX1	4222	0	down
FOXP2	93986	0	down
SOBP	55084	0	down
SLC6A9	6536	0	down
TACR1	6869	0	down
BTK	695	0	up
NLGN4X	57502	0	up
ZMAT3	64393	0	up
ATF7IP2	80063	0	up
P2RY12	64805	0	up
MR1	3140	0	up
CHD7	55636	0	up
CHPF	79586	0	up
SLC15A3	51296	0	up
SLC46A3	283537	0	up

DEmRNAs, differentially expressed mRNAs; FDR, false discovery rate.

**Table 4:** All DEmiRNAs between patients with VS and normal controls.

Symbol	FDR	Regulation
hsa-mir-340	0.000965	down
hsa-mir-10b	0.000965	down
hsa-mir-625	0.002215	down
hsa-mir-182	0.002215	down
hsa-mir-412	0.004423	down
hsa-mir-183	0.004423	down
hsa-mir-181a	0.00834	down
hsa-mir-206	0.008414	down
hsa-mir-126	0.009816	down
hsa-mir-34a	7.66E-09	up
hsa-mir-30a	0.000965	up
hsa-mir-9	0.000965	up
hsa-mir-21	0.000965	up
hsa-mir-601	0.002215	up
hsa-mir-184	0.002215	up
hsa-mir-628	0.00404	up
hsa-mir-654	0.00404	up
hsa-mir-30c	0.008414	up
hsa-mir-106b	0.009057	up
hsa-mir-377	0.009057	up
hsa-mir-22	0.009412	up

DEmiRNAs, differentially expressed miRNAs; FDR, false discovery rate.

## Data Availability

The data used to support the findings of this study are included within the article.
